# Novel *TSPAN12* mutations in patients with familial exudative vitreoretinopathy and their associated phenotypes

**Published:** 2011-04-29

**Authors:** Huiqin Yang, Xueshan Xiao, Shiqiang Li, Guiying Mai, Qingjiong Zhang

**Affiliations:** State Key Laboratory of Ophthalmology, Zhongshan Ophthalmic Center, Sun Yat-sen University, Guangzhou, P.R. China

## Abstract

**Purpose:**

Mutations in tetraspanin 12 (*TSPAN12*) have recently been identified as a cause of autosomal dominant familial exudative vitreoretinopathy (FEVR). The purpose of this study was to detect *TSPAN12* mutations in Chinese patients with FEVR and to describe the associated phenotypes.

**Methods:**

Sanger sequencing was used to analyze the seven coding exons and their adjacent regions of *TSPAN12* in 49 unrelated FEVR patients. Clinical phenotypes of the patients with *TSPAN12* mutations were documented.

**Results:**

Three novel heterozygous mutations in *TSPAN12* were identified in three patients from unrelated families: c.146C>T (p.Thr49Met), c.313T>C (p.Cys105Arg), and c.601delC (p.Leu201PhefsX14). All three mutations involved highly conserved residues and were not present in 180 normal individuals. Ocular phenotypes included retinal folds, inferotemporal dragging of the optic disc and macula, increased vessels in the equatorial region, and a peripheral avascular zone. A father and his daughter had the same mutation but the father only had mild peripheral fundus changes while his daughter had obvious dragged disc and macular ectopia.

**Conclusions:**

Our results suggest that *TSPAN12* mutations are responsible for FEVR. Similar to patients with mutations in *NDP*, *LRP5*, or *FZD4*, the phenotypes associated with *TSPAN12* mutations showed great variations between different individuals within a family and between the two eyes in individual patients.

## Introduction

Familial exudative vitreoretinopathy (FEVR, OMIM 133780) is a hereditary vitreoretinal disorder characterized by developmental anomalies of the retinal vessels [1]. The primary anomaly is a premature arrest of the vascularization in the peripheral retina, which may lead to retinal neovascularization, vitreoretinal traction, exudates, fibrovascular masses, vitreous hemorrhages, retinal folds, and tractional retinal detachment. FEVR exhibits strikingly variable phenotypes among patients, even for patients from the same family or between the two eyes of an individual patient. The most severe form results in complete blindness, whereas minimally affected individuals can be totally asymptomatic, in which nonperfusion or avascular zones in the peripheral retina may only be identified by fluorescein angiography [2].

FEVR can be inherited as an autosomal dominant (OMIM 133780) [1,3-5], autosomal recessive (OMIM 601813) [6,7], or X-linked (OMIM 305390) [8,9] trait, and the autosomal dominant form is the most common [10,11]. Mutations in *NDP* (OMIM 300658) [8,12], *FZD4* (OMIM 604579) [13], and *LRP5* (OMIM 603506) [14,15] have been reported to be responsible for FEVR. The proteins encoded by these genes have all been shown to participate in the Wnt/Norrin signaling pathway [16-19]. Mutations in *NDP*, *FZD4*, and *LRP5* account for approximately half of all FEVR cases, which indicates that additional causative genes remain to be identified [20-26].

Recently, heterozygous mutations in tetraspanin 12 (*TSPAN12*; OMIM 613138), which is a component of the Norrin-FZD4-LRP5 signaling complex [16,19], have been found to be responsible for autosomal dominant FEVR [27,28]. However, the frequency of *TSPAN12* mutations and their associated phenotypes need further study.

In this study, Sanger sequencing was used to analyze the coding and adjacent intronic regions of *TSPAN12* in 49 unrelated Chinese FEVR patients. Three novel heterozygous mutations were identified, and the associated phenotypes were described.

## Methods

### Patients

Probands with FEVR from 49 unrelated families were collected at the Pediatric and Genetic Eye Clinic, Zhongshan Ophthalmic Center, Guangzhou, China. Of the 49, 34 were sporadic and 15 had a family history of FEVR. Clinical diagnosis of FEVR was based on the presence of at least one of the following clinical findings that suggested primary retinal vascular developmental defects as previously described [21,23,24]: a peripheral retinal avascular zone with or without fibrous proliferation, peripheral neovascularization showing increased branching or a brushlike border, a peripheral fibrovascular mass, temporal dragging of the optic disc and/or macula, straightening of the temporal retinal vessels, falciform retinal folds, tractional retinal detachment with or without retinal exudates or vitreous hemorrhages, or total retinal detachment with fibrotic mass behind the lens. Retinal fluorescein angiography was performed in some suspicious cases to confirm the diagnosis of FEVR. Patients with a possible diagnosis of retinopathy of prematurity were excluded from the study. This study was approved by the Internal Review Board (IRB) of the Zhongshan Ophthalmic Center. It complied with the guidelines of the Declaration of Helsinki and the Guidance of Sample Collection of Human Genetic Diseases (863-Plan) of the Ministry of Public Health of China. Informed consent was obtained from the participating individuals or their guardians before the collection of clinical data and genomic samples.

### Detection of *TSPAN12* mutations

Genomic DNA was retrieved from our genomic DNA repository, which was established for the genetic study of hereditary eye disease. Seven pairs of primers (Table 1) were designed to amplify all the coding exons and the adjacent intronic sequences of *TSPAN12* (reference sequences from NCBI: NC_000007.13 for gDNA, NM_012338.3 for mRNA, and NP_036470.1 for protein). Touchdown PCR was performed, with the annealing temperature decreased by 2 °C after the first 5 cycles and the second 5 cycles, and then down to the optimal annealing temperature (listed in Table 1) for the remaining 25 cycles. The sequences of the amplicons were determined with an ABI BigDye Terminator Cycle Sequencing Kit, v3.1, using an ABI 3100 Genetic Analyzer (Applied Biosystems, Foster City, CA). Sequences from patients and *TSPAN12* consensus sequences from the NCBI human genome database (NC_000007.13) were compared using the SeqManII program of the Lasergene package (DNAstar, Madison, WI). Each variation was initially confirmed by bidirectional sequencing and then evaluated in 180 normal individuals (360 chromosomes). The description of the mutations was based on the recommendations of the Human Genomic Variation Society. The effect of a missense mutation on the encoded protein was predicted by the PolyPhen-2 online tool [29].

**Table 1:** Primers used for PCR amplification and sequencing of *TSPAN12*.

**Exon**	**Forward primer(5′-3′)**	**Reverve primer(5′-3′)**	**Amplicon size (bp)**	**Annealing tempeture (°C)**
2	GCTGTGGGAAGCGTGAT	TTAGCCATGCCCTTTGG	281	58
3	TTCAAGATGCAGCAAATGGTAAT	GCTATGGGCAGGAAAAACTT	329	58
4	ATGTCTTGGGTGCATTT	AAGCGTCCCTTCTTACA	321	56
5	AGGGCTCACTGAGATGA	TCTTGGGCAGTTCTTTC	382	55
6	TTAGAAGACATTCCGAGTA	AATTGCACCAGAGGTTA	360	55
7	TCCTTTACTACATTTCTATC	TAGCTTTCTTCTGCTTC	462	52
8	ACAGATTGTTTGCTTTC	AGGTGTTATTTTATGGC	505	52

## Results

**Figure 1:**
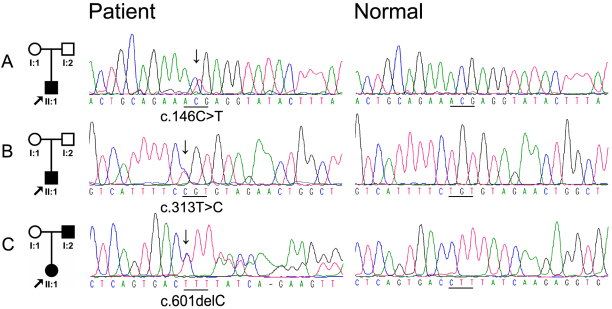
Mutations identified in *TSPAN12*. **A**: The patient in the family had c.146C>T mutation. **B**; The patient in the family had c.313T>C mutation. **C**: The affected father and daughter had c.601delC mutation. The columns from left to right display the pedigree and the sequence chromatograms for these patients and the normal controls.

After sequencing the coding and adjacent regions of *TSPAN12* in 49 unrelated FEVR patients, three novel heterozygous mutations were detected in three patients: c.146C>T, c.313T>C, and c.601delC (Figure 1). These three mutations were not present in the 180 normal individuals.

**Figure 2:**
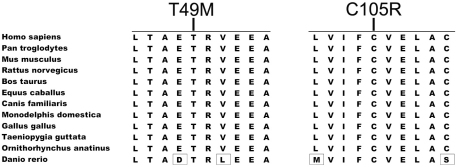
Protein alignment of 12 (*TSPAN12*) orthologs demonstrates conservation in the regions with mutations. The 12 orthologs are from the following 12 species: *Homo sapiens* (NP_036470), *Pan troglodytes* (XP_001142304), *Mus musculus* (NP_766595), *Rattus norvegicus* (Q569A2), *Bos taurus* (NP_001039977), *Equus caballus* (XP_001502093), *Canis familiaris* (XP_855095), *Monodelphis domestica* (XP_001364876), *Gallus gallus* (NP_001007850), *Taeniopygia guttata* (XP_002192381), *Ornithorhynchus anatinus* (XP_001516347), and *Danio rerio* (NP_957446). The regions with the two missense mutations are highly conserved. T49M stands for p.Thr49Met and C105R stands for p.Cys105Arg.

The c.146C>T mutation in exon 3 changed the encoded residue from a hydrophilic threonine to a sulfur-containing hydrophobic methionine at codon 49 (p.Thr49Met), which involves a highly evolutionarily conserved residue (Figure 2). This mutation was predicted to probably be damaging by PolyPhen-2. The c.313T>C mutation in exon 5 changed the encoded residue from a thiol-containing cysteine to a positively charged basic arginine at codon 105 (p.Cys105Arg), which is highly conserved (Figure 2). This mutation was also predicted to probably be damaging by PolyPhen-2. The c.601delC mutation in exon 7 not only changed the residue at codon 201, but also created a frame shift with 14 additional new residues before a premature termination at codon 215 (p.Leu201PhefsX14). This mutation also involved an evolutionarily conserved region.

**Figure 3:**
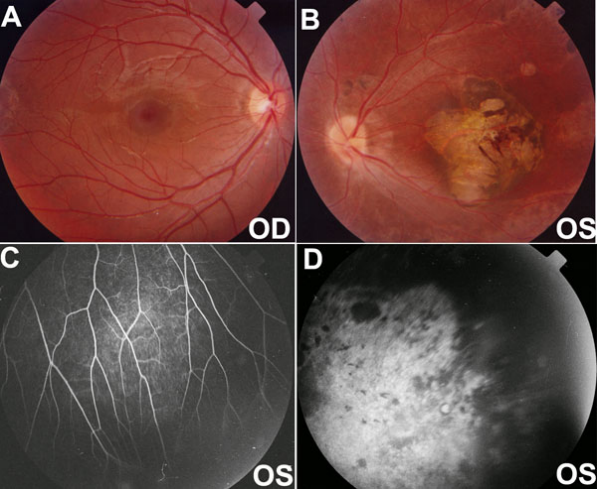
Fundus changes in the patient with the heterozygous c.313T>C mutation in Family B. **A** and **B**: These color photos demonstrate a normal right posterior fundus and traction of the retinal vessels and macular degeneration in the left fundus. **C** and **D**: Fluorescein angiography of the left eye shows straightening of the vessels with increasing branching (**C**) and a peripheral avascular zone (**D**). OD and OS represent the right and left eyes, respectively.

**Figure 4:**
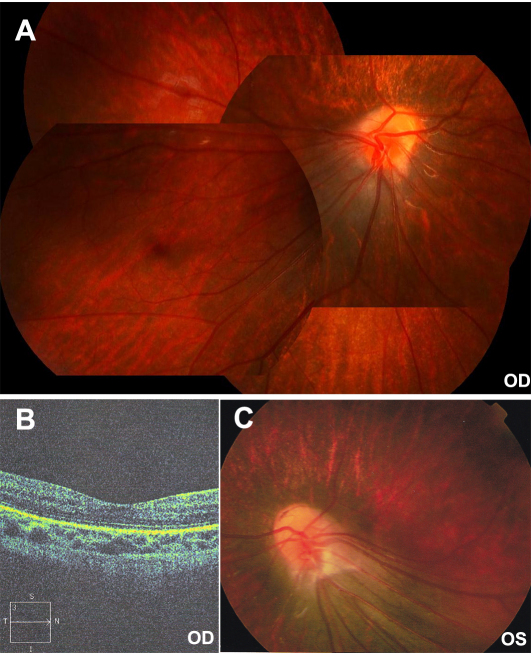
Fundus changes in the proband with the c.601delC mutation in Family C. **A**: Inferotemporal dragging of the optic disc and macula is present in the right eye. **B**: Optical coherence tomography scan shows a flatter central macula in the right eye. **C**: Inferotemporal dragging of the optic disc and macula is present in the left eye. OD and OS represent the right and left eyes, respectively.

**Figure 5:**
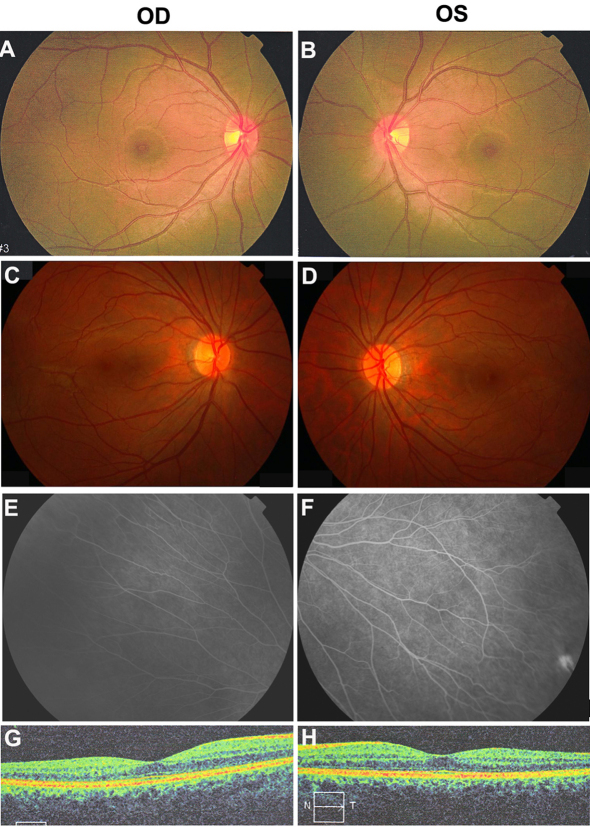
Fundus photos of the asymptomatic father with the c.601delC mutation and the unaffected mother without the mutation. **A** and **B**: The mother has normal fundi. **C**-**D**: Fundus photos of the asymptomatic father shows normal posterior fundi. **E** and **F**: The father has increased vessel branching in the equatorial area and a peripheral avascular zone. **G** and **H**: Optical coherence tomography scan shows normal macula of the asymptomatic father.

All patients with the three novel mutations showed typical signs of FEVR. The patient with the c.146C>T mutation in Family A was a six-year-old boy with strabismus. Falciform retinal folds in the left fundus were seen upon ophthalmoscopic observation. His parents were recorded to have normal visual acuity, but they were not available for additional examination. The patient with the c.313T>C mutation in Family B was a nine-year-old boy with a complaint of poor visual acuity in the left eye. He had normal visual acuity in the right eye but only recognized hand movement with the left eye. His right fundus was normal, but his left fundus showed the following changes: macular degeneration with pigment proliferation, mild temporal dragging of the optic disc, pigmentary deposits on the midperipheral retina, increased ramification of the peripheral retinal vessels, and an avascular zone on the peripheral retina (Figure 3). His parents were recorded to have normal visual acuity, but they were not available for additional examination. The heterozygous c.601delC mutation in Family C was detected in the proband and in her asymptomatic father. The proband was a three-year-old girl with a complaint of esotropia. Fundus examination showed typical inferotemporal dragging of the optic disc and macula in both eyes (Figure 4). Her mother had normal fundi (Figure 5A,B); but her asymptomatic father had typical fundus changes, although he had normal visual acuity of 1.0 in both eyes. The asymptomatic father had straightening and increased branching of his peripheral retinal vessels (Figure 5C-F) but showed normal macula on optical coherence tomography scan (Figure 5G,H).

## Discussion

In this study, three novel *TSPAN12* mutations were detected in three patients with FEVR, but not in 180 normal individuals. Segregation analysis in one family indicated a dominant role for the mutation. The two missense mutations occurred in evolutionarily conserved regions of *TSPAN12* and were predicted to be pathogenic. These lines of evidence provide support for the conclusion that these mutations are the cause of FEVR in these patients.

**Figure 6:**
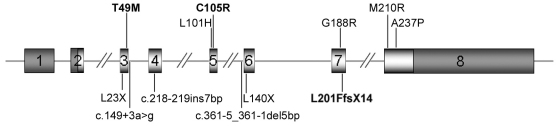
Schematic representation of *TSPAN12* and its familial exudative vitreoretinopathy mutations. Missense mutations are shown above the gene and others below the gene. The 12 mutations listed in this figure included the three from our study (letters in bold) and nine from two previous reports [27,28]. The dark shaded parts in exons 1, 2, and 8 represent the untranslated regions. The vertical lines in exons 2 and 8 indicate the positions of translation start and terminate, respectively.

Previously, nine *TSPAN12* mutations in patients with FEVR were reported. All 12 mutations, including the three reported in this study, are distributed throughout all coding exons, except for exon 2 (Figure 6). These 12 mutations can be classified into missense mutations (six), nonsense mutations (two), insertion or deletion mutations (two), and splice-site mutations (two). Of the 12 mutations, each has only been found in a single family, except for the p.Ala237Pro mutations that have been detected in four families. For the 15 families with the 12 *TSPAN12* mutations, ten had a family history of FEVR, which suggests that this is an autosomal dominant trait with incomplete penetrance, whereas the other five families were isolated cases without a family history. The approximately 6.1% (3/49) mutation frequency detected in our patients is comparable to the 10% (7/70) frequency detected in one previous study [28]. The patients in that study were determined to have no mutations in the other known FEVR genes (*NDP*, *FZD4*, or *LRP5*) that might account for approximately half of FEVR cases [30]. In another study, two *TSPAN12* mutations were present in 5 of 11 probands (45.5%), but these patients were previously excluded from having mutations in *NDP*, *FZD4* or *LRP5.* Four of the five probands with the same mutation shared an at-risk haplotype of at least 5.8 Mb, suggesting a founder effect [27].

For the patients with the previously reported *TSPAN12* mutations, clinical phenotypes include the following: retinal folds, temporal dragging of the optic disc and/or macula, traction of the posterior retinal vessels, increased vascular ramifications in the equatorial area, a peripheral retinal avascular zone, retinal pigmentary disturbances that mimic retinitis pigmentosa, exudates, and retinal detachments. Retinal folds were the most common finding. FEVR in asymptomatic individuals can be detected using fluorescein angiography (Figure 5), but mild changes in the peripheral retina may be neglected, which might partly contribute to the nonpenetrance in a few families. Large phenotypic variations have been observed between different individuals within a family and between different eyes in individual patients. There is no particular genotype–phenotype correlation between particular mutations and certain clinical signs, which suggests haploinsufficiency as the underlying mechanism that induces the manifestation of the disease. Overall, the ocular manifestations caused by *TSPAN12* mutations are similar to those due to mutations in *FZD4*, *LRP5*, or *NDP* [21,23,24].

The proteins encoded by *TSPAN12*, *NDP*, *FZD4*, and *LRP5* are important components of the Norrin/Fz4 signaling pathway (Norrin/Fz4/Lrp5/Tspan12 signaling pathway), which controls retinal vascular development [16,19]. Recognizing additional mutations in these genes may not only provide useful information for clinical diagnosis and genetic counseling, but may also enrich our understanding of the functional domains of these proteins. Mutations in these four genes all lead to FEVR, which suggests that additional components that participate in this signaling pathway may be reasonable candidates in those FEVR patients without mutations in these four genes.
